# Genome-wide transcriptome analysis and drug target discovery reveal key genes and pathways in thyroid cancer metastasis

**DOI:** 10.3389/fendo.2025.1514264

**Published:** 2025-02-10

**Authors:** Minjing Zou, Amal Qattan, Monther Al-Alwan, Hazem Ghebeh, Naif Binjumah, Latifa Al-Haj, Khalid S. A. Khabar, Abdulmohsen Altaweel, Falah Almohanna, Abdullah M. Assiri, Abdelilah Aboussekhra, Ali S. Alzahrani, Yufei Shi

**Affiliations:** ^1^ Department of Molecular Oncology, King Faisal Specialist Hospital and Research Centre, Riyadh, Saudi Arabia; ^2^ Department of Cell Therapy and Immunobiology, King Faisal Specialist Hospital and Research Centre, Riyadh, Saudi Arabia; ^3^ Department of Molecular Biomedicine, King Faisal Specialist Hospital and Research Centre, Riyadh, Saudi Arabia; ^4^ Department of Comparative Medicine, King Faisal Specialist Hospital and Research Centre, Riyadh, Saudi Arabia; ^5^ Department of Medicine, King Faisal Specialist Hospital and Research Centre, Riyadh, Saudi Arabia

**Keywords:** CD274 (PD-L1), TBXAS1, MERTK, IL6, thyroid cancer metastasis

## Abstract

**Introduction:**

Metastasis is the major cause of thyroid cancer morbidity and mortality. However, the mechanisms are still poorly understood.

**Methods:**

We performed genome-wide transcriptome analysis comparing gene expression profile of metastatic thyroid cancer cells (Met) with primary tumor cells established from transgenic mouse models of papillary thyroid cancer (PTC), follicular thyroid cancer (FTC), poorly differentiated thyroid cancer (PDTC), and anaplastic thyroid cancer (ATC).

**Results:**

Genes involved in tumor microenvironment (TME), inflammation, and immune escape were significantly overexpressed in Met cells. Notably, IL-6-mediated inflammatory and PD-L1 pathways were highly active in Met cells with increased secretion of pro-inflammatory and pro-metastatic cytokines such as CCL2, CCL11, IL5, IL6, and CXCL5. Furthermore, Met cells showed robust overexpression of Tbxas1, a thromboxane A synthase 1 gene that catalyzes the conversion of prostaglandin H2 to thromboxane A2 (TXA2), a potent inducer of platelet aggregation. Application of aspirin, a TXA2 inhibitor, significantly reduced lung metastases. Mertk, a member of the TAM (Tyro, Axl, Mertk) family of RTKs, was also overexpressed in Met cells, which led to increased MAPK activation, epithelial–mesenchymal transition (EMT), and enrichment of cancer stem cells. Braf-mutant Met cells developed resistance to BRAFV600E inhibitor PLX4720, but remained sensitive to β-catenin inhibitor PKF118-310.

**Conclusion:**

We have identified several overexpressed genes/pathways in thyroid cancer metastasis, making them attractive therapeutic targets. Given the complexity of metastasis involving multiple pathways (PD-L1, Mertk, IL6, COX-1/Tbxas1-TXA2), simultaneously targeting more than one of these pathways may be warranted to achieve better therapeutic effect for metastatic thyroid cancer.

## Introduction

Thyroid cancer is the most common malignancy in the endocrine system and its incidence have been rising in the past few decades with vast majority of this increase being ascribed to papillary thyroid carcinoma (PTC) (1–3). The rise in incidence seems to be due to over-diagnosis, but an actual increase in incidence and mortality cannot be completely ruled out (2, 4). The follicular cell-derived thyroid cancer can be histologically classified into papillary thyroid carcinoma (PTC), follicular thyroid carcinoma (FTC), poorly differentiated thyroid carcinoma (PDTC) and anaplastic thyroid carcinoma (ATC). Differentiated thyroid carcinoma (DTC) has excellent prognosis with a 10-year disease-specific survival of up to 90%, PDTC has poorer prognosis with a 5-year disease-specific survival at 66%. ATC is highly virulent with a mean survival of less than 8 months (5–7). PTC is the most common type of DTC, accounting for more than 85% of all thyroid cancer cases followed by FTC (5-10%), PDTC (4-7%) and ATC (about 2%) (7, 8).

The initiation and progression of thyroid cancer occur through gradual accumulation of multiple genetic alterations, leading to constitutive activation of two crucial signaling pathways: MAPK and PI3K/AKT (9). MAPK activation is considered to be crucial for PTC initiation, through point mutations of *BRAF* and *RAS* genes or chromosomal rearrangements of *RET/PTC* and neurotrophic tropomyosin receptor kinase (*NTRK*) gene. The *BRAF^V600E^* mutation is the most frequent genetic alteration in PTC with overall rate of 48.5% (10). PI3K/AKT activation is critical in FTC initiation and can be triggered by activating mutations in *RAS*, *PIK3CA*, and *AKT1* as well as by inactivation of *PTEN*. *RAS* (*HRAS*, *KRAS* and *NRAS*) mutations occur in 30–45% of FTC (11). The progression and dedifferentiation to PDTC and ATC are thought to arise from preexisting DTC as a result of acquiring additional genetic changes such as mutations in *TERT* promoter, *TP53*, *EIF1AX*, and *CDKN2A.TP53* mutations are present in 50–80% of ATC and is one of the pivotal molecular alterations discriminating ATC from PTC or FTC (12).

Distant metastasis is the leading cause of thyroid cancer mortality and morbidity (13, 14). The metastatic cascade represents a multi-step process which includes local tumor cell invasion of the basement membrane, intravasation into the vasculature, survival in the circulation, extravasation from the circulation, and final colonization by the circulating tumor cells at the distal sites (15). Distant metastasis occurs in about 10% of PTC and up to 25% FTC patients. The most common distant metastatic sites are lungs (~80%) and bones (~25%) (16, 17). The five-year survival rate dropped from 77.6% to 15.3% in patients with single organ and multi-organ distant metastasis, respectively (14).

Activation of metastatic reprogramming and survival of circulating tumor cells in the blood with eventual colonization at distant organs are critical steps for thyroid cancer metastasis. These steps are influenced by both tumor intrinsic (genetic mutations and epigenetic modifications) and extrinsic factors (tumor microenvironment or TME) (5, 16). However, detailed mechanisms contributing to thyroid cancer metastasis are still lacking. Understanding the underlying mechanisms would enable targeted intervention. The present study investigated the mechanisms that regulate circulating tumor cell survival in the blood circulation and colonization at lung by using cell lines derived from genetically engineered mouse models of PTC, FTC, PDTC, and ATC, representing the whole spectrum of thyroid carcinogenesis.

## Materials and methods

### Experimental animals

Athymic BALB/c-nu/nu (nude mice) were acquired from Jackson Laboratory. Mice were provided with autoclaved food and water ad libitum. The study was approved by the Animal Care and Use Committee of the institution and was conducted in compliance with the Public Health Service Guidelines for the Care and Use of Animals in Research (RAC#2230003).

### Thyroid cancer cell lines

Four murine thyroid cancer cell lines derived from genetically engineered mouse models of PTC, FTC, PDTC, and ATC were established from primary tumors: PTC with *Braf* ^V600E^ mutation (BVE), FTC with *Kras* ^G12D^ mutation (KGD), PDTC with both *Kras^G12D^* and *Cdkn2a^null^* mutations (KGD^Cdkn2a_null^), and ATC with both *Braf* ^V600E^ and *Trp53* ^null^ mutations (BVE^Trp53_null^). The establishment of BVE, KGD, and BVE^Trp53_null^ strains were described previously (18–21). KGD^Cdkn2a_null^ was established by cross-breeding among *Kras^G12D^*, TPO-Cre, and *Cdkn2a^null^* (strain 01XE4 obtained from The NCI Mouse Repository, (https://frederick.cancer.gov/resources/data-repositories/nci-mouse-repository) (79). PDTC was developed from a 13-month-old mouse with both *Kras^G12D^* and *Cdkn2a^null^* mutations. The KGD^Cdkn2a_null^ cell line was established from the tumor. Thyroid origin was confirmed by genotyping (**Supplementary Figure 1**). The cell lines were maintained in DMEM/F12 growth medium containing 10% fetal bovine serum, 100 units/ml penicillin, and 100 μg/ml streptomycin.

### Metastatic thyroid cancer cell lines

**Figure 1:**
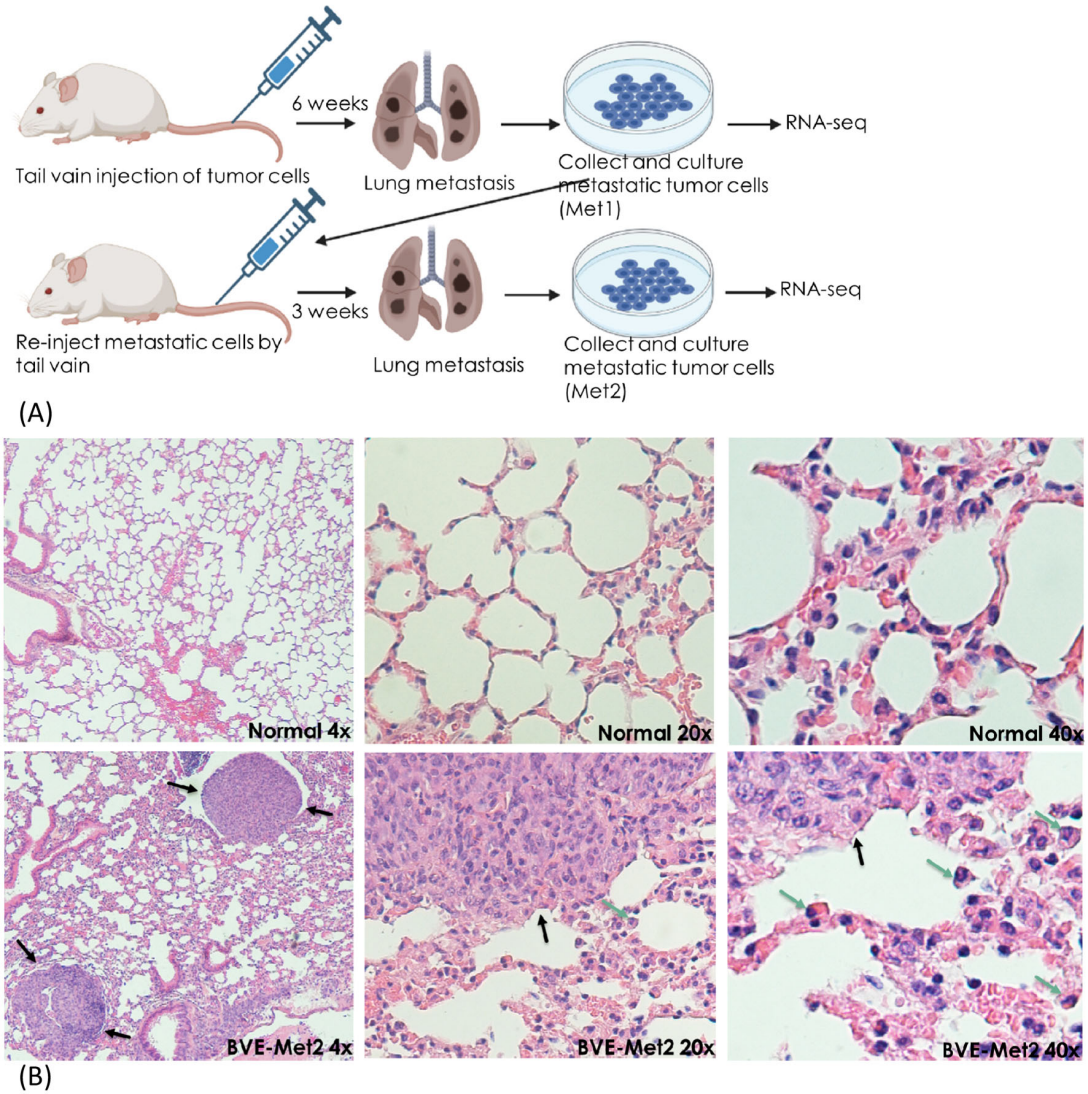
Experimental lung metastasis. **(A)** Schematic diagram summarizing experimental lung metastatic procedure. BVE, BVE^Trp53null^, KGD, or KGD^Cdkn2anull^ cells (1 x 10^6^ cells) were injected to tail vain of 5 nude mice for each group. Six weeks after injection, lung metastatic tumors were collected to establish Met1 cell lines. The established Met1 cells were re-injected (1 x 10^6^ cells) to a new group of nude mice (n=5 for each group) via tail vain for enrichment of cells with high metastatic potential. Lung metastatic tumors were harvested three weeks following injection to establish Met2 cell lines. Met1, Met2 and primary tumor cells were subject to RNA sequencing to identify differentially expressed genes (DEGs). **(B)** Lung metastatic foci after tail vain injection of BVE thyroid cancer cells (H&E staining). Metastatic foci are indicated by black arrows and tumor infiltrating monocytes and macrophages are indicated by green arrows.

To establish lung metastatic thyroid cancer cell lines, 1 x 10^6^ BVE, KGD, KGD^Cdkn2a_null^ or BVE^Trp53_null^ cells were injected to tail vain of 5 nude mice for each group. Six weeks after injection, lung metastatic tumors were collected aseptically from the mice using blunt dissection, then mechanically dissociated by mincing and passing through a 40-μM mesh sterile screen, and suspended in DMEM/F12 growth medium for 3 months with a total of 6 passages to eliminate contaminated stromal fibroblasts, lymphocytes, and microphages present in the tumor cell culture. The primary culture were considered as permanent cell lines after six passages. The established metastatic cell lines were named as BVE-Met1, KGD-Met1, KGD^Cdkn2a_null^-Met1, and BVE^Trp53_null^-Met1, respectively. They were re-injected (1 x 10^6^ cells) to a new group of nude mice (n=5 for each group) via tail vain for enrichment of cells with high metastatic potential. Three weeks following injection, lung metastatic tumors were harvested and propagated in DMEM/F12 growth medium for 3 month with at least 6 passages. The metastatic cell lines were named as BVE-Met2, KGD-Met2, KGD^Cdkn2a_null^-Met2, and BVE^Trp53_null^-Met2, respectively. The experimental procedures were summarized in **Figure 1A** and representative lung metastatic foci were presented in **Figure 1B**. The thyroid origin of these cell lines were confirmed by genotyping.

### RNA sequencing analysis

RNA-Seq were used for quantification of differentially expressed genes (DEGs) between primary (BVE, KGD, KGD^Cdkn2a_null^, and BVE^Trp53_null^) and metastatic thyroid cancer cell lines: BVE-Met1, KGD-Met1, KGD^Cdkn2a_null^-Met1, and BVE^Trp53_null^-Met1, or BVE-Met2, KGD-Met2, KGD^Cdkn2a_null^-Met2, and BVE^Trp53_null^-Met2 cell lines. Total RNA from cell lines were isolated and libraries were constructed using an Illumina (San Diego, Ca, USA) TruSeq RNA Library Prep kit according to the manufacturer’s procedure. Sequencing was performed on Illumina HiSeq 4000 with at least 20 million clean reads. The significant DEGs were selected based on the following criteria: Log2-fold change >2, false discovery rate (FDR) <0.001, and P-value from difference test<0.01. Gene list annotation and enrichment of biological pathways were performed using Metascape (https://metascape.org/gp/index.html#/main/step1).

### The protein-protein interaction network construction and hub genes identification

Interactions among different proteins were performed using NetworkAnalyst (https://networkanalyst.ca/) to analyze an interactive relationship among DEGs. Genes with the degree of a node >10 (with more than 10 interacting genes) were considered as hub genes. The association of hub gene expression with disease-specific survival was performed by Kaplan–Meier analysis using TCGA-THCA mRNA expression dataset (n=498) and cBioPortal For Cancer Genomics (https://www.cbioportal.org/).

### Quantitative real-time reverse transcriptase-PCR

qRT-PCR was used to validate DEGs as described previously (21). The cDNA mix was diluted 10-fold, and 2 μl of the dilution was used for qPCR analysis. The PCR conditions were 94°C for 30 sec followed by 30 cycles of amplification (94°C for 10 sec, 55°C for 5 sec, and 72°C for 10 sec). The PCR primers were listed below: Cd274-F, 5’-ACGGTGGTGCGGACTACAAG-3’(exon 3) and Cd274-R: 5’-TCCAGATTACCTCAGCTTCT-3’ (exon4); Tbxas1-F, 5’-AGAGCCAATTGGAACTCCGAG-3’ (exon 3), Tbxas1-R, 5’-ACCTGCTTGATCATGTCTGG-3’ (exon4); Mertk-F, 5’-CAGCTGGCATTTCATGGTGGAA-3’ (exon2), Mertk-R, 5’-TTCATCTTACAGAAGTACGAC-3’ (exon 3). The resulting concentration of target PCR products was normalized by comparison with β-actin and was used to determine the relative mRNA level of DEGs in Met2 cells.

### Cytokine/chemokine measurements

Tumor cells were cultured for 48h and the conditioned media were collected for cytokine and chemokine measurement using MILLIPLEX Mouse Cytokine/Chemokine panel coupled with the Luminex^®^ xMAP^®^ platform according to manufacturer’s instruction (EMD Millipore Corporation, Billerica, MA). The following 32 cytokines and chemokines were measured simultaneously: Eotaxin (CCL11), G-CSF (CSF3), GM-CSF (CSF2), IFN-γ, IL-1α, IL-1β, IL-2, IL-3, IL-4, IL-5, IL-6, IL-7, IL-9, IL-10, IL-12 (p40), IL-12 (p70), IL-13, IL-15, IL-17, IP-10 (CCL10), KC (CXCL1), LIF(Leukemia inhibitory factor), LIX (CXCL5), MCP-1 (CCL-2), M-CSF (CSF1), MIG (CXCL9), MIP-1α (CCL3), MIP-1β (CCL4), MIP-2 (CXCL2), RANTES (CCL5), TNF-α, and VEGF.

### Western blot analysis

Cell lysates were obtained by extraction in RIPA buffer (20mM Tris-HCl, pH7.4, 150mM NaCl, 5 mM EDTA, 1% NP-40) containing Pierce’s Halt Protease Inhibitor Cocktail (Thermo Scientific, Rockford, IL). Proteins (40 µg) were loaded onto a 12% SDS-polyacrylamide gel and were transferred to a PVDF membrane. Western blot analysis was performed using antibodies (1:1000 dilution) from Cell Signaling Technology (Danvers, MA) against phospho-Erk (#4370, RRID: AB_2315112), phosphor-Akt (#4060, RRID: AB_2315049), E-Cadherin (#3195, RRID: AB_2291471), and Vimentin (#5741, RRID: AB_10695459), or antibodies against IL-6 (ab281935, RRID: AB_3661729), IL-6 receptor (ab300582, RRID: AB_300582), and PD-L1 (ab269674) from Abcam (Boston, MA). The experiments were repeated twice.

### Flow cytometry analysis for cell surface markers and apoptosis

The expression of Ep-CAM, CD11b, CD24, and CD44 cell surface markers on tumor cells was analyzed by FACS (fluorescence-activated cell sorting) flow cytometer (LSR I; Becton Dickinson, Mountain View, CA, USA) using anti-Ep-CAM-APC (Biolegend, San Diego, CA, USA, CAT#118214, RRID: AB_1134102), anti-CD11b (BD Biosciences, Heidelberg, Germany, CAT# 552850, RRID: AB_394491), anti-CD24-APC (Biolegend, CAT# 101814, RRID: AB_439716) and anti-CD44-PE-Cy7 (Biolegend, CAT#103030, RRID: AB_830787) labelled antibodies. The CSC-like cell subpopulation was identified by gating on CD44^high^/CD24^low^ cells, while differentiated-like cells were identified as CD44^low^/CD24^high^ cells (22). Ep-CAM was used to identify epithelial cells and epithelial cell-derived tumor cells, while CD11b was used to mark myeloid-lineage cells such as monocytes/macrophages, neutrophils cells. BVE and BVE-Met2 cells were cultured with different concentrations of PKF118-310, PLX4720, or both for 24h. The apoptosis was analyzed using the Vybrant apoptosis assay kit (Molecular Probes, Eugene, OR, USA).

### Preparation and administration of aspirin to mice

The mouse dose equivalent to 100–150 mg/60kg human low dose aspirin was calculated as human equivalency dose (HED) = animal dose (mg/kg) × (animal km)/(human km), where mouse km factor is 3, and human km factor is 37. Aspirin (Chewable Aspirin 81 mg, Bayer) was prepared at 2.5 mg/mL in PBS to administer 10 µl/g body weight to deliver a dose of 25 mg/kg. Mice (n=10) were injected by tail vain of 1 x 10^6^ BVE^Trp53_null^-Met2 cells for lung metastasis. They were divided into 2 groups: group 1 (n=5) were given aspirin 3 times/week by oral gavage for 4 weeks while control mice (group 2) received the same volume of PBS.

### Colony formation assay

The sensitivity of BVE and BVE-Met2 cells to BRAF^V600E^ and β-catenin inhibitors were determined by colony formation assay. BVE and BVE*-*Met2 cells were plated into 6-well plates with different low cell seeding number (50 to 200 cells/well) for 14 days in the presence or absence of different concentrations of PLX4720, PKF118-310 or both. Cells were then fixed with methanol for 10 min and stained with 0.5% crystal violet dye in methanol:de-ionized water (1:5) for 10 min. After three washes with H2O to remove excess crystal violet dye, colonies containing more than 50 individual cells were counted using a microscope. Three separate experiments were performed and average were presented. Colony forming efficiency (CFE) was calculated using the formula: CFE = (number of colonies counted/number of cells plated) × 100.

### Sphere formation assay

Tumor sphere assay was performed as described previously (23). Briefly, thyroid tumor cells (10000 cells/ml) were cultured in ultra-low attachment plates (Corning) in DMEM-F12 (Life Technologies) containing stem cell culture supplements (4% FBS, 1% antibiotics, 1% glutaMax, 2% B-27, 20 ng/ml EGF, 20 ng/ml bFGF, 500 ng/ml hydrocortisone, 5 µg/ml insulin, and 2 U/ml heparin). After 10 days in culture, spheres >50 μm were counted. Three separate experiments were performed and average were presented.

### Statistical analysis

Student’s *t*-test (two-tailed) was used to compare two groups and one-way ANOVA was used to compare multiple groups. A *P* value of 0.05 or less was considered significant.

## Results

### Genome-wide transcriptome analysis to identify critical genes in thyroid cancer metastases

**Figure 2:**
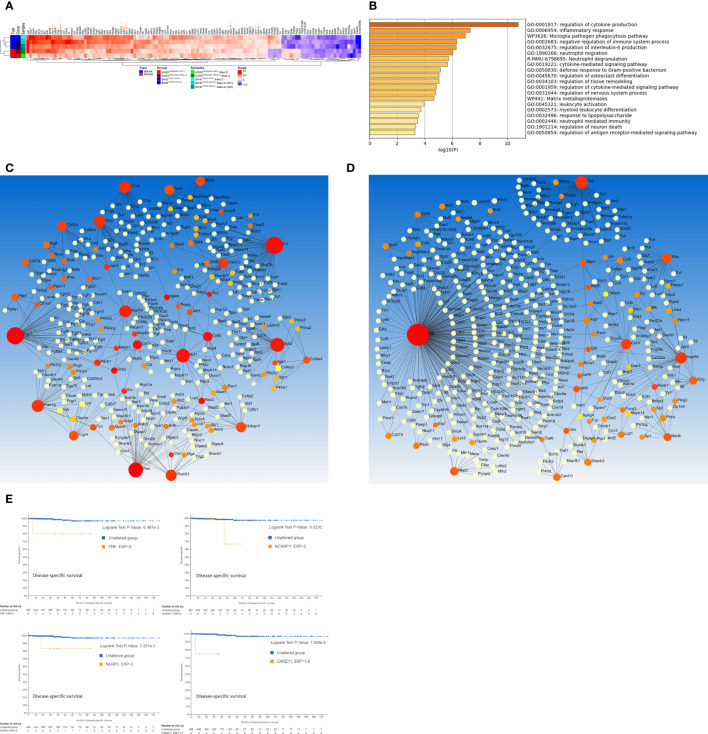
Genome-wide transcriptome analysis of DEGs in Met2 cells. **(A)** Heatmap of DEGs present in all 4 Met2 cells (BVE-Met2, BVE^Trp53null^-Met2, KGD-Met2, and KGD^Cdkn2a_null^-Met2). Several key genes involved in inflammation, immune checkpoint regulation and cancer stem cells are indicated by an arrow. **(B)** Top 20 Enriched ontology clusters of DEGs by Metascape analysis. Regulation of cytokine production, inflammatory response, phagocytosis pathway, negative regulation of immune system, and regulation of IL6 production are the top 5 enriched pathways. **(C)** PPI network and hub genes identification by NetworkAnalyst. STRING interactome with high confidence score of 900 (of maxium1000) was chosen for hub gene identification. Eighteen genes with the degree of a node >10 (bigger red nodes) were selected as hub genes **(D)** PPI network based on InnateDB. Six genes with the degree of a node >10 (bigger red nodes) were selected as hub genes. **(E)** Kaplan-Meier analyses of hub gene expression on disease-specific survival of PTC patients. Overexpression of 4 hub genes (Tnf, Nckap1, Nlrp3, and Card11) was associated with poor disease-specific survival. TCGA-THCA mRNA dataset (n=498) was used for Kaplan-Meier analysis.

To identify common genes and pathways driving thyroid cancer metastasis, we performed a comprehensive gene expression profile of primary and Met cell lines carrying single oncogenic driver mutations (*Braf* ^V600E^ or *Kras* ^G12D^) or in combination with inactivation of tumor suppressor genes (*Braf* ^V600E^ and *Trp53* ^null^ or *Kras^G12D^* and *Cdkn2a^null^*). Many differentially expressed genes (DEGs) that were not detected in the Met1 cells appeared in the Met2 cells (**Supplementary Figure 2**). Additionally, Met2 cells formed lung metastases 3 weeks faster than Met1 cells (3 vs 6 weeks), indicating enrichment of tumor cells with high metastatic potential. Transcriptome analysis identified 88 up-regulated and 22 down-regulated genes (log2 fold-change >2) present in all four Met2 cell lines (**Figure 2A**; **Supplementary Table 1**). Gene ontology and pathway analysis showed that regulation of cytokine production, inflammatory and negative regulation of immune response, neutrophil migration and phagocytosis, and cytokine- or receptor-mediated pathways were the top enriched ontology clusters (**Figure 2B**). PPI network analysis by STRING identified 18 hub genes: *Tnf* (degree of node 47), *Fgfr1* (46), *Was* (30), *Itgb2* (27), *Ncf1* (25), *Fcer1g* (20), *C1qa* (19), *Inpp5d* (19), *Il2rg* (18), *Shank3* (15), *Nckap1* (13), *Nod1* (13), *Card11* (12), *Csf2rb* (12), *Cybb* (11), *Fcgr4* (11), *Nlrp3* (11), *Csf1r* (10) (**Figure 2C**). Consistent with STRING results, 5 were also identified as hub genes by InnateDB (innate immunity database): *Tnf* (degree of node 41), *Inpp5d* (28), *Csf1r* (20), *Was* (18), and *Nlrp3* (10) (**Figure 2D**). Intriguingly, *Irf8*, the most significant hub gene (with degree of node 272) was not identified by the STRING. The detailed function of these hub genes and their biological roles in cancer metastasis were listed in **Supplementary Table 2**. We next analyzed the association of these hub gene over-expression with disease-specific survival of PTC patients using thyroid carcinoma dataset (TCGA, PanCancer Atlas) (n=498). The over-expression of 4 hub genes (*Tnf*, *Nckap1*, *Nlrp3*, and *Card11*) was associated with poor disease-specific survival (**Figure 2E**). These 4 genes are involved in the inflammation and/or epithelial-mesenchymal transition (EMT) pathways, indicating the critical role of these pathways in thyroid cancer metastases, and may be useful biomarkers to predict disease prognosis.

### Increased production of inflammatory cytokines by metastatic tumor cells

**Figure 3:**
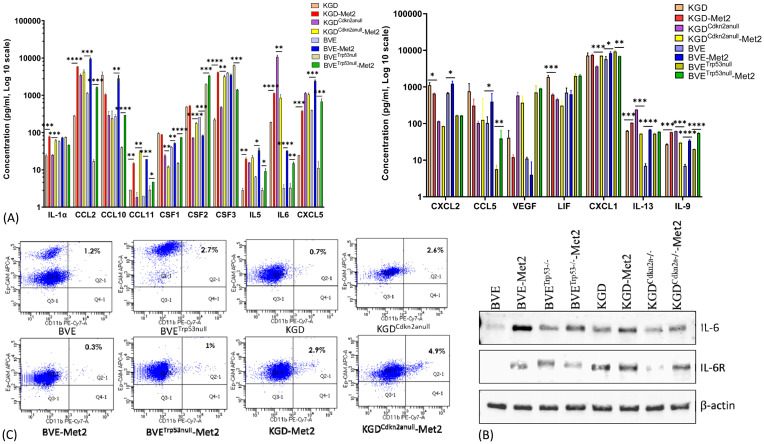
Cytokine/Chemokine production by Met2 cells. **(A)** Cytokine/Chemokine production was detected in conditioned media of both primary and Met2 cells using MILLIPLEX mouse ELISA assay. Increased production of inflammatory cytokines and chemokines were observed in Met2 cells. **(B)** Western blot analysis of IL-6 and IL-6 receptor expression in primary and Met2 cells. Increased IL-6 and IL-6 receptor expression were observed in Met2 cells. **(C)** FACS analysis of Ep-CAM and CD11b staining of both primary and Met2 cells. Ep-CAM is an epithelial cell marker, while CD11b is a myeloid cell marker highly expressed on the surface of innate immune cells, including macrophages and neutrophils. CD11b-positive cells were present in less than 5% of Met2 cell population *P<0.05, **P<0.01, ***P<0.001.

To assess whether over-expression of above genes in Met2 cells correlated with more inflammatory cytokine/chemokine production, we analyzed 32 cytokine and chemokine levels in the conditioned media of both primary and Met2 cells. As shown in **Figure 3A**, Met2 cells produced more inflammatory cytokines or chemokines such as CSF1 (colony stimulating factor 1 or macrophage colony-stimulating factor), CSF2 (colony-stimulating factor 2 or granulocyte-macrophage colony-stimulating factor), CSF3 (colony-stimulating factor 3 or granulocyte colony-stimulating factor), CCL2 (monocyte chemoattractant protein-1), CCL11 (eosinophil chemotactic protein), CXCL5 (epithelial-derived neutrophil-activating peptide 78, a chemotactic chemokine known to activate neutrophil during acute inflammatory responses), IL-1α and IL-6. We further confirmed increased expression of IL-6 and/or IL-6 receptor in Met2 cells by Western blot (**Figure 3B**). These cytokines/chemokines have been reported to promote tumor cell immune escape and metastasis by recruitment of tumor-associated macrophages (TAMs), myeloid-derived suppressor cells (MDSCs), tumor-associated neutrophils (TANs), and regulatory T (Treg) cells (24). Consistent with the reported roles of these cytokines/chemokines in mobilizing immunosuppressive inflammatory cells, there were increased monocyte and macrophage infiltration in TME (**Figure 1B**) and overexpression of Arg1 (arginase1), an immunosuppressive signal found predominantly on TAMs, in Met2 cells (**Figure 2A**; **Supplementary Table 1**). It has been shown that breast cancer-derived CSF2 regulates Arg1 to promote an immunosuppressive TME (25). Primary and Met2 cells were also analyzed by FACS for the expression of Ep-CAM (epithelial cell marker) and CD11b (myeloid cell marker highly expressed on the surface of monocytes/macrophages, and some CD8+ cytotoxic T cells) to rule out the possibility that increased inflammatory cytokine/chemokine production observed in the Met2 cells was due to contaminating monocytes, macrophages, and tumor-infiltrating lymphotyes. As shown in **Figure 3C**, majority of tumor cells were Ep-CAM^+^ with minimal CD11b^+^, indicating that Met2 cells were epithelial origin without significant contaminating monocytes/macrophages. The small fraction of CD11b^+^ cells (<5%) may be due to increased EMT in Met2 cells.

### Identification of druggable genes and pathways in metastatic tumor cells

**Figure 4:**
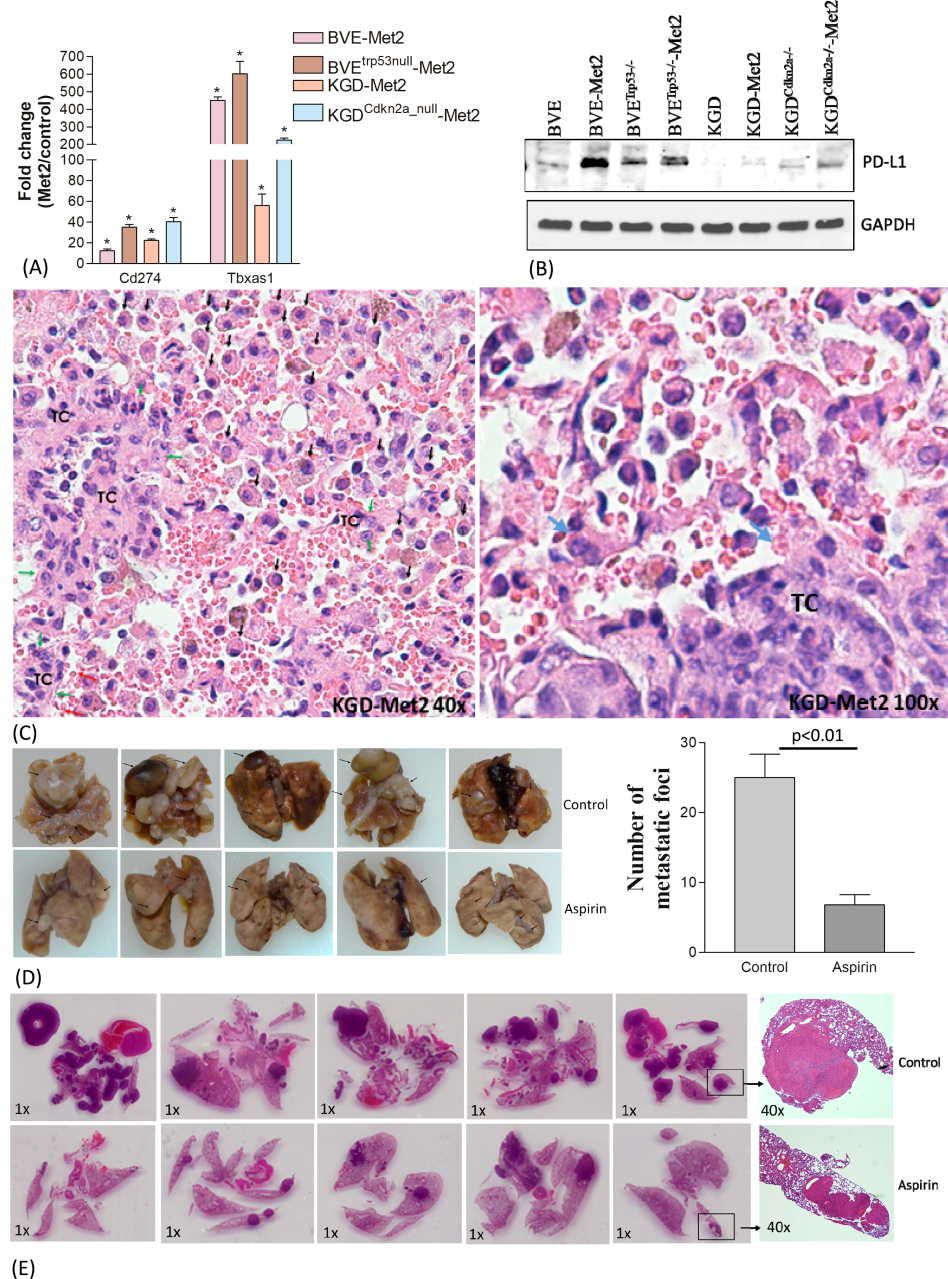
Contribution of Cd274 and Tbxas1 in pulmonary pre-metastatic niche formation. **(A)** Overexpression of Cd274 (PD-L1) and Tbxas1 mRNA in Met2 cells as compared to primary cells detected by qRT-PCR analysis.*p<0.01. **(B)** Western blot analysis of CD274 expression. Increased CD274 expression were observed in Met2 cells. **(C)** Microscopic metastatic foci of KGD-Met2 cells. Significant infiltration of RBCs, lymphocytes, and macrophages are noted at the interface between metastatic tumor foci and lung. Tumor cells are indicated by green arrows and marked as TC. Increased infiltration of monocytes and macrophages is indicated by black arrows. Increased RBC aggregation is noted and platelets aggregation on tumor cells is indicated by a blue arrow. **(D)** Lung metastasis of BVE^Trp53null^-Met2 upon low dose aspirin treatment. The metastatic foci on the lung surface were counted and plotted as a bar graph. Significant reduction of lung metastatic foci was observed when mice were given aspirin (25 mg/kg) by oral gavage for 4 weeks. Data are presented as mean ± SEM. Representative metastatic foci are indicated by an arrow. **(E)** Histology (H&E staining) of lung metastases of thyroid tumor cells. The size and number of metastatic foci were reduced after aspirin treatment.

**Figure 5:**
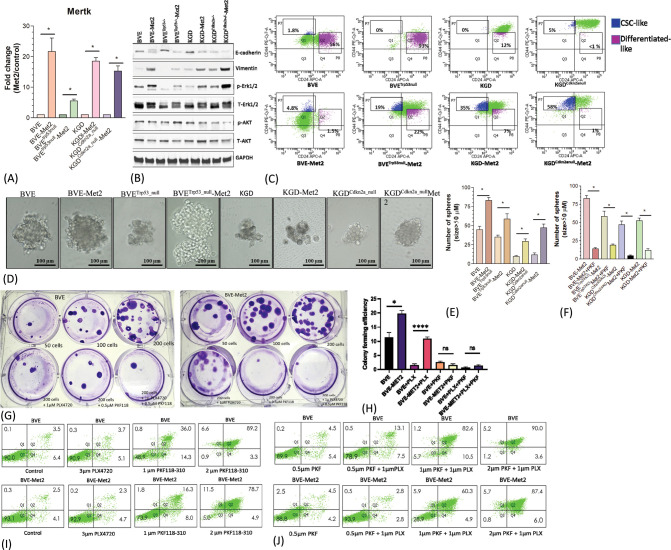
Up-regulation of MerTK-mediated signaling. **(A)** Mertk mRNA overexpression in Met2 cells detected by qRT-PCR analysis. *p<0.01. **(B)** Western blot analysis of E-cadherin, vimentin, p-Erk, and p-AKT expression in Met2 cell lines. Increased expression of vimentin and p-Erk and decreased expression of E-cadherin were demonstrated in all Met2 cells. Increased p-AKT expression was present only in the BVE^trp53null^-Met2 cells. **(C)** FACS analysis of CD44/CD24 expression in Met2 cells. Increased CD24^low^CD44^high^ staining was observed in Met2 cells. **(D)** Tumorspheres formation in ultralow attachment stem cells culture conditions. Representative tumorspheres are shown. **(E)** The number of spheres was counted and plotted as a bar graph. The number of tumorspheres was increased in Met2 cells, *p<0.01. **(F)** Inhibition of tumorspheres by PKF118-310. Significant reduction of tumorspheres was noted upon treatment with 1µM PKF118-310, *p<0.01. **(G)** Sensitivity of BVE-Met2 cells to BRAF^V600E^ and β-catenin inhibitors. BVE and BVE-Met2 cells were cultured in different low cell seeding numbers. A representative colony formation assay was shown. **(H)** The number of colonies was counted and plotted as a bar graph. Increased colony formation was observed in low seeding number of BVE-Met2 cells, which were resistant to BRAF^V600E^ inhibitor PLX4720 but remained sensitive to β-catenin inhibitor PKF118-310. *P<0.05, ****P<0.0001, NS, not statistically significant. **(I)** Flow cytometric analysis of apoptotic BVE and BVE-Met2 cells after treatment with BRAF^V600E^ or β-catenin inhibitors. **(J)** Flow cytometric analysis of apoptotic BVE and BVE-Met2 cells after combined treatment of BRAF^V600E^ and β-catenin inhibitors.

The approach to identify common DEGs present in all 4 Met2 cell lines irrespective of their underlying genetic defects yielded encouraging results above. This strategy significantly reduced the number of DEGs to be analyzed from more than 2200 in each Met2 cell line to 110 (**Figure 2A**; **Supplementary Figure 2**), which increased the efficiency to identify drug targets and decreased false identification. We then focused on these genes to identify drug targets that could be applicable to all 4 tumor types. Tumor cell-induced platelet aggregation is a well-recognized mechanism for paraneoplastic thrombocytosis and a potential cause of reduced response to immune checkpoint inhibitors in hematogenous metastasis. Tbxas1, a thromboxane A synthase 1 gene which catalyzes the conversion of prostglandin H2 to thromboxane A2 (TXA2), was highly expressed in Met2 cells (**Figures 2A**, **4A**). TXA2 is a potent inducer of platelet aggregation. The overexpression of Tbxas1 would trigger coagulation cascade and may explain the significant RBC infiltration observed in pre-metastatic niche of lung metastases (**Figure 4C**). Importantly, treatment with a low-dose of aspirin, an irreversible inhibitor of TXA2, significantly reduced the size and number (6.8 ± 1.5 vs 25 ± 3.4, p<0.01) of lung metastatic foci after intravenous injection of BVE^Trp53_null^-Met2 cells to nude mice (n=5) (**Figures 4D, E**), thus, confirming the role of Tbxas1in promoting platelet aggregation and metastatic niche formation. Moreover, Met2 cells over-expressed not only two immune checkpoint regulators, CD274 (PD-L1) (**Figures 4A, B**) and CD52 (**Figure 2A**, **Supplementary Figure 2**), but also Mertk, a member of the TAM (Tyro, Axl, Mertk) family of receptor tyrosine kinases (**Figures 2A**, **5A**). Mertk has been reported to activate multiple signaling pathways (JAK/STAT, MAPK, PI3K/AKT, EMT, and PD-1/PD-L1) to promote tumor cell migration, immune escape, and cancer stem cell (CSC) transformation, and act as bypass mechanisms to EGFR blockade (26). Indeed, up-regulation of several down-stream signaling targets of Mertk was observed in Met2 cells such as increased expression of PD-L1 (**Figure 4B**), increased expression of vimentin and decreased expression of E-cadherin (EMT↑), and increased p-Erk (MAPK↑) (**Figure 5B**). Increased expression of p-AKT was found only in BVE^Trp53null^-Met2 cells (**Figure 5B**). Taken together, these data strongly suggest the contribution of these genes/pathways to immune escape of metastatic tumor cells.

### Metastatic tumor cells retain features of CSCs and resistance to monotherapy with BRAF^V600E^ inhibitor PLX4720

The role of WNT/μ-catenin pathway in thyroid cancer progression is well established (27). Met2 cells showed over-expression of Limb-Bud- and-Heart (LBH) (**Figure 2A**; **Supplementary Table 1**), a WNT/β-catenin target required for normal mammary stem cell self-renewal (28) and an oncogene specifically expressed in tumor-initiating CD24^low^/CD44^high^ breast CSCs with high metastatic potential (29). Given Mertk is also involved in CSC transformation, we investigated CD44 and CD24 expression in Met2 cells. As shown in **Figure 5C**, increased CD44 and decreased CD24 expression was observed in Met2 cells, indicating either increased transformation to CSCs or enrichment of CSCs. Tumorsphere and colony formation are two *in vitro* stemness functional assays. Met2 cells showed more tumorsphere formation (**Figures 5D, E**) that was significantly reduced (more than 70% as compared to the control) after treatment with 1 µM β-catenin inhibitor PKF118-310 (**Figure 5F**). Only 10% reduction in tumorsphere formation was observed in BVE-Met2 and no reduction in BVE^Trp53null^-Met2 cells upon treatment with 2 µM of BRAF^V600E^ inhibitor PLX4720 (**Supplementary Figure 4**), indicating resistance to BRAF^V600E^ inhibitor. In the colony formation assay, BVE-Met2 cells showed increased formation of colonies at low seeding numbers (19.7 ± 1.2 *vs* 11.3 ± 1.7, p<0.05) and resistance to 1 µM BRAF^V600E^ inhibitor PLX4720 (10.8 ± 0.7 vs 1.5 ± 0.3 colonies, p<0.01) (**Figures 5G, H**). Both BVE and BVE-Met2 cells remained sensitive to 0.5 µM of β-catenin inhibitor PKF118-310 (11.3 ± 1.7 in BVE control vs 2.5 ± 0.3 colonies in BVE treatment, p<0.01; 19.7 ± 1.2 in BVE-Met2 control vs 1.5 ± 0.3 in BVE-Met2 treatment, p<0.01) (**Figures 5G, H**). Furthermore, as compared to 3 µM PLX4720 alone, PLX4720-induced apoptosis was significantly enhanced by combination of 1 µM PLX4720 and 1 µM PKF118-310: 8.8% vs 93.1% in BVE cells, and 7% vs 65.2% in BVE-Met2 cells (**Figures 5I, J**). Collectively, metastatic thyroid tumor cells expressed stemness features and became more resistance to BRAF^V600E^ inhibitor PLX4720, but were still sensitive to β-catenin inhibitor PKF118-310. Combinational therapy with low doses of PLX4720 and PKF118-310 showed synergistic effect in BVE-Met2 cells.

## Discussion

**Figure 6:**
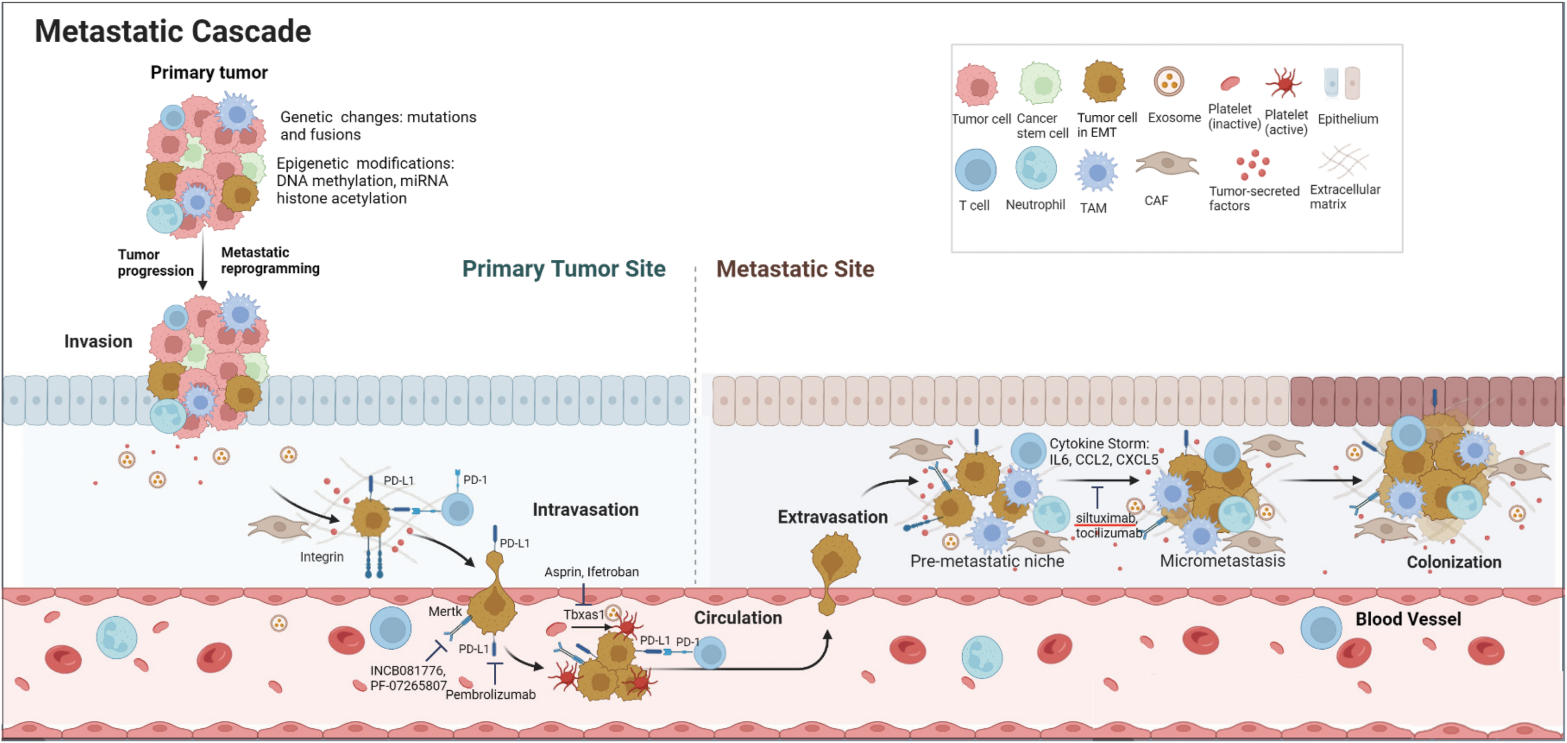
Schematic diagram of metastatic cascade of thyroid cancer cells and potential drug targets. Genetic changes and epigenetic modifications drive tumor progression and metastatic reprogramming. To evade immune elimination and survive in the blood circulation, metastatic cells express high levels of ‘Don’t eat me’ signals such as Cd274 (PD-L1) and Cd52 as well as Tbxas1 and Mertk. At the metastatic site, the metastatic cells induce a cytokine storm by secreting a large amount of inflammatory cytokines and chemokines to help form a pre-metastatic niche and eventual colonization at the distant site.

In the present study, we investigated the transcriptome landscape of metastatic tumor cells from 4 different thyroid cancer transgenic mouse models ranging from well-differentiated (PTC and FTC) to poorly-differentiated (PDTC) and anaplastic thyroid cancer (ATC). Despite different genetic mutations driving oncogenic transformation, we identified a group of metastatic genes and pathways shared by all four tumor types. The simultaneous activation of several key genes and pathways involved in endoplasmic reticulum (ER) stress and inflammation, platelet aggregation, negative immune regulation, and Mertk receptor tyrosine kinase provided insight into how metastatic cells evade immune elimination, survive in the circulation, and finally colonize at distant sites. The metastatic process and potential drug targets are illustrated in **Figure 6**.

Tail vein injection may not be the ideal method for studying metastasis mechanisms because it bypasses the first two steps of metastasis process (tumor cell invasion of the basement membrane and intravasation into the vasculature). Successful colonization of the secondary organ is the rate-limiting step in the metastatic process. However, since mouse models of thyroid cancer do not develop spontaneous metastases, we used tail-vein injection methods to interrogate key factors required for cancer cell colonization of secondary organs. This approach has been used for a high-throughput *in vivo* screening method in the mouse for identifying regulators of metastatic colonization (30) and is still valid for studying how tumor cells survive in the circulation, extravagate from the circulation, and colonize at the distal organs.

Currently, the main goal of anti-cancer therapies relies on the effective elimination of cancer cells by apoptosis (31). Paradoxically, cell-death-inducing therapies can enhance metastasis by inducing changes in TME triggered by a cell-death-driven cytokine storm and tumor-associated macrophages (32–36). Conod et al. have demonstrated that tumor cells that survive impending death become stable pro-metastatic tumor cells. These cells exhibit features such as metastatic reprogramming, stemness, cytokine storm, and ER stress, which could induce neighboring tumor cells to acquire pro-metastatic states and form distant metastases *in vivo* (37). Indeed, Met2 cells demonstrated these features such as stemness, increased inflammatory cytokine production, and ER stress. In a recent study of anaplastic transformation in thyroid cancer, Lu et al. uncovered the spectrum of ATC transformation: inflammatory PTC cells (iPTCs) → inflammatory ATC cells (iATCs) → mesenchymal ATC cells (mATCs), indicating inflammation plays an important role in ATC transformation (38). They further demonstrated that mATCs contributed to poor overall survival with aneuploid genomes, mesenchymal phenotypes, and overexpression of collagen genes (i.e., COL1A1, COL1A2, COL3A1, COL5A1, COL5A2) (38). Interestingly, Col6a3, a member of collagen family of genes, was over-expressed in Met2 cells and associated with poor disease-specific survival in PTC patients (TCGA-THCA dataset, **Supplementary Figure 3**).

NADPH oxidase isoform 2 (NOX2) is a multicomponent enzyme complex including 5 subunits: Cyba, Cybb, Ncf1, Ncf2, and Ncf4 and expressed almost solely in myeloid cells such as monocytes, macrophages and neutrophilic granulocytes. It generates reactive oxygen species (ROS) in defense against microbial pathogens. A recent study has showed it promotes melanoma pulmonary metastasis by inhibiting adjacent lung-infiltrating cytotoxic T and NK cells (39, 40). Two of NOX2 subunits (Ncf1 and Cybb) were highly expressed in Met2 cells, which may induce ER stress in TME and promote pulmonary metastatic colonization. The increased Ncf1 and Cybb expression in Met2 may reflect EMT and metastatic reprograming.

PD-L1 expression was elevated in all Met2 cells. Given nude mice has no functional T cells, the immune evasion is likely mediated by inhibition of NK cells. PD-1/PD-L1 blockade enhances anti-tumor efficacy of NK cells (41, 42). Various signaling pathways regulate PD-1/PD-L1 expression. PI3K/AKT, MAPK, JAK/STAT, Wnt/β-catenin, NF-κB, and Hedgehog pathways have all been reported to increase the expression of PD-1/PD-L1 axis (43). PD-L1 is highly glycosylated in tumor cells to maintain its stability via IL-6/JAK1-mediated phosphorylation and subsequent glycosylation (44). Since IL6 is a pro-inflammatory cytokine known to promote cancer metastasis and induces PD-1 expression in activated T cells, blocking IL6 pathway may reduce tumor immune escape in TME and enhance anticancer immunity (44). IL6 antibody siltuximab, IL6 receptor antibody tocilizumab, JAK1/2 kinase inhibitor ruxolitinib, and PD-L1 antibody (Atezolizumab, Avelumab, Durvalumab) have been approved by the FDA, simultaneously targeting both PD-1/PD-L1 and IL6/JAK1 may be more effective than single agent in cancer immunotherapy (45, 46).

Barkal et al. have recently identified CD24, a glycosylphosphatidylinositol (GPI)-anchored protein, as a novel ‘don’t eat me’ signal expressed on tumor cells to evade macrophage-mediated phagocytosis by binding to Siglec10 inhibitory receptor expressed on tumour-associated macrophages (47). Although CD24 overexpression was not found in Met2 cells, another GPI-anchored protein CD52 was highly expressed in all Met2 cells, which binds to the same inhibitory receptor Siglec10 to suppress immune cell function (48, 49). The Siglec10 is widely expressed in immune cells, such as B cells, monocytes, dendritic cells, NK cells, and a subset of activated T cells. CD52 may function as a novel ‘don’t eat me’ signal and potential drug target.

Tumor-secreted cytokines and chemokines play an important role in promoting cancer metastasis. CCL2, a potent chemokine in macrophage recruitment and polarization during inflammation was produced abundantly in Met2 cells. CCL2-secreting breast cancer cells have been shown to interact with CCR2+ macrophages to facilitate their metastasis to lung and bone (50). Similarly, CCL3 (macrophage inflammatory protein-1 α), a pro-inflammatory chemokine implicated in tumor metastasis, was highly expressed in Met2 cells. The CCL3-CCR5 axis regulates intratumoral trafficking of leukocytes and fibroblasts to promote angiogenesis and subsequent lung metastasis (51). IL6 production was elevated in Met2 cells (52). Tumor cell-secreted IL6 has been reported to induce CCL5 expression in lymphatic endothelial cells and accelerate metastasis in triple-negative breast cancer (53).

Platelets contribute to tumor metastasis via multiple mechanisms at different stages of metastatic cascades (54). Met2 cells expressed high levels of Tbxas1 for platelet activation. Activated platelets are involved in the formation of metastatic niche, which could be inhibited by aspirin (55). The benefit of aspirin in reducing cancer metastasis has been confirmed in clinical trials (56). The current study provides a mechanistic explanation for targeting COX-1/Tbxas1-TXA2 pathway against metastatic thyroid cancer. It has been reported that platelets could increase PD-L1 expression on tumor cells via NF-κB and TGFβ signaling (57). Furthermore, PD-L1 protein can be transferred from tumor cells to platelets to suppress anti-tumor immune response (58). The transfer of PD-L1 protein from tumor cells to platelets may explain the high levels of PD-L1 transcripts and subtle increase of PD-L1 protein in Met2 cells. The platelet aggregation and transfer of PD-L1 to platelets would create a physical and molecular shield to protect tumor cells. Thus, targeting COX-1/TXA2 signaling may improve the efficacy of PD-L1 immunotherapy by disrupting this protective shield. Given unwanted gastrointestinal side effects of aspirin, a potent and selective TXA2 receptor antagonist, Ifetroban, has been re-evaluated for the treatment of metastatic cancer and is currently under Phase II clinical trial (NCT03694249) (59).

One of the significant findings reported in this study was Mertk overexpression in Met2 cells. Mertk is known to activate multiple signaling pathways (MAPK, PI3K/AKT, JAK/STAT, and PD-1/PD-L1) in many types of cancer to promote immune tolerance, tumor progression and metastasis, and drug resistance (26, 60, 61). It likely plays a pivotal role in Met2 cell resistance to BRAF inhibitor and survival of NK cell-mediated immune elimination. Mertk signaling in tumor cells could increase PD-L1 expression to foster immune escape and survival (62). Paolino et al. have reported TAM inhibition enhances NK cell activity, leading to markedly reduction in murine mammary cancer and melanoma metastases (63). They have further showed that low-dose warfarin, which inhibits TAM receptor activity without affecting coagulation, exerts anti-metastatic activity in mice via Cbl-b/TAM receptors on NK cells (63, 64). Several Axl/Mer inhibitors, such as INCB081776 (NCT03522142) and PF-07265807 (NCT04458259), are currently under Phase I clinical trial in patients with advanced or metastatic solid tumors (65).

In summary, we have uncovered several key genes and pathways driving thyroid cancer metastasis. They act in synchrony to promote inflammation, cytokine storm, platelet aggregation, and subsequently pre-metastatic niche formation. Given that cancer cells utilize multiple pathways to evade immune elimination and extensive cross-interactions among these pathways, combinational therapy against PD-L1, Tbsax1, and/or IL-6 may offer better therapeutic outcome.

## Data Availability

The datasets presented in this study can be found in online repositories. The names of the repository/repositories and accession number(s) can be found in the article/**Supplementary Material.**
