# Supplementary material for: Genome-wide transcriptome analysis and drug target discovery reveal key genes and pathways in thyroid cancer metastasis
Source: Front Endocrinol (Lausanne). 2025 Feb 10;16:1514264. doi: 10.3389/fendo.2025.1514264 (PMC11847698; doi:10.3389/fendo.2025.1514264)
Supplement: Supplementary file 1 [file DataSheet1.pdf]

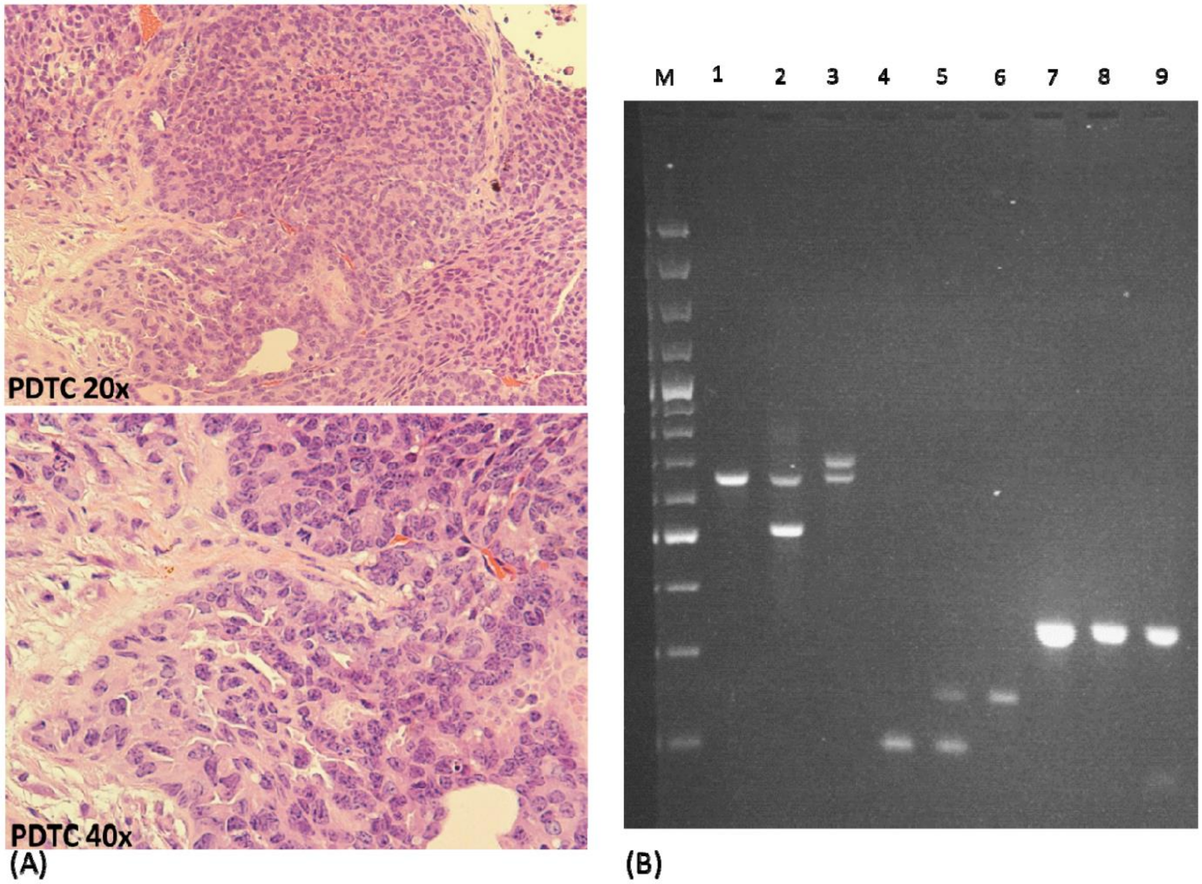

Supplementary Figure 1. Poorly differentiated thyroid cancer (PDTC) with both *Kras*^{G12D} and *Cdkn2a*^{null} mutations from 13-month-old mouse. (A) Histology of PDTC. (B) Genotyping of PDTC cell line derived from the primary tumor: M, 1kb ladder; lane 1, Wt Kras from a control mouse tail; lane 2, LSL-*Kras*$^{G12D/+}$ from a control mouse tail (not activated by Cre-mediated recombination); lane 3, LSL-*Kras*$^{G12D/+}$ activated from the PDTC cell line by Cre-mediated recombination; lane 4, Wt *Cdkn2a* from a control mouse tail; lane 5, Cdkn2a$^{flox/flox}$ from a control mouse tail; lane 6, *Cdkn2a* deleted from PDTC cell line by Cre-mediated recombination; lane7, Cre from a control Wt *Kras* mouse tail; lane 8, Cre from a control LSL-*Kras*$^{G12D/+}$ mouse tail; lane 9, Cre from a control *Cdkn2a*$^{flox/flox}$ mouse tail.

Symbol	KGD -Met1	KGD^{Cdkn2a_null}-Met1	BVE-Met1	BVE^{Trp53_null} -Met1	KGD-Met2	KGD^{Cdkn2a_null} -Met2	BVE-Met2	BVE^{Trp53_null} -Met2
CD274	ND	2.8 (7)	ND	3.59 (12)	2.62 (6)	4.39 (21)	5.08 (34)	6.45 (87)
CD47	1.66 (3.17)	ND	ND	ND	2.14 (4.4)	ND	2.24 (4.7)	ND
B2M	1.4 (2.6)	ND	2.1(4.2)	ND	2.1(4.3)	1.2 (2.3)	3.6 (11.8)	ND
CD24	ND	-1.9 (-3.8)	-6 (-64)	-3.8 (-14.4)	ND	-5 (-31.8)	-4 (-16.4)	-4.3 (-19.4)
CD52	4.25 (19)	1.85 (3.6)	ND	ND	5.7 (52)	7.7 (20)	12 (4400)	6.5 (653)
TBXAS1	ND	3.91 (15)	ND	3.7 (13)	5.36 (41)	7.89 (238)	9.2 (598)	10.3 (1285)

Supplementary Figure 2. ‘Don’t eat me’ signal expression in Met1 and Met 2 cells. Log2 fold change vs control of DEGs (simple fold change vs control in parentheses). Cd274 (PD-L1) overexpression was not uniformly found in Met1 cells (not detected in KGD-Met1 and BVE-Met1 cells), but was present and enriched in all Met2 cells. Similar pattern was observed for Cd52 and Tbxas1, indicating immune elimination of metastatic cancer cells with lower expression of Cd274, Cd52, and Tbxas1 by NK cells. Strictly speaking, Tbxas1 is not a “Don’t eat me’ signal”, but it indirectly helps cancer cells evade immune elimination via platelets activation and aggregation.

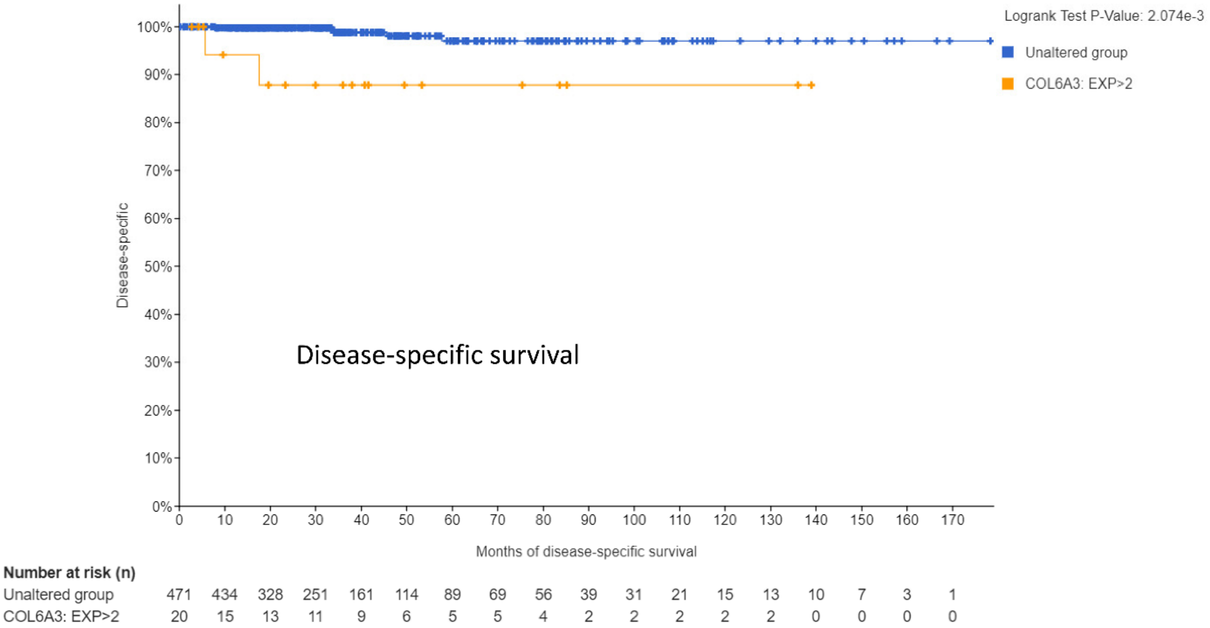

Supplementary Figure 3. Kaplan-Meier plot showing disease-specific survival of PTC patients (n=499, TCGA-THCA mRNA expression dataset). Kaplan–Meier analysis was performed using cBioPortal For Cancer Genomics (https://www.cbioportal.org/).

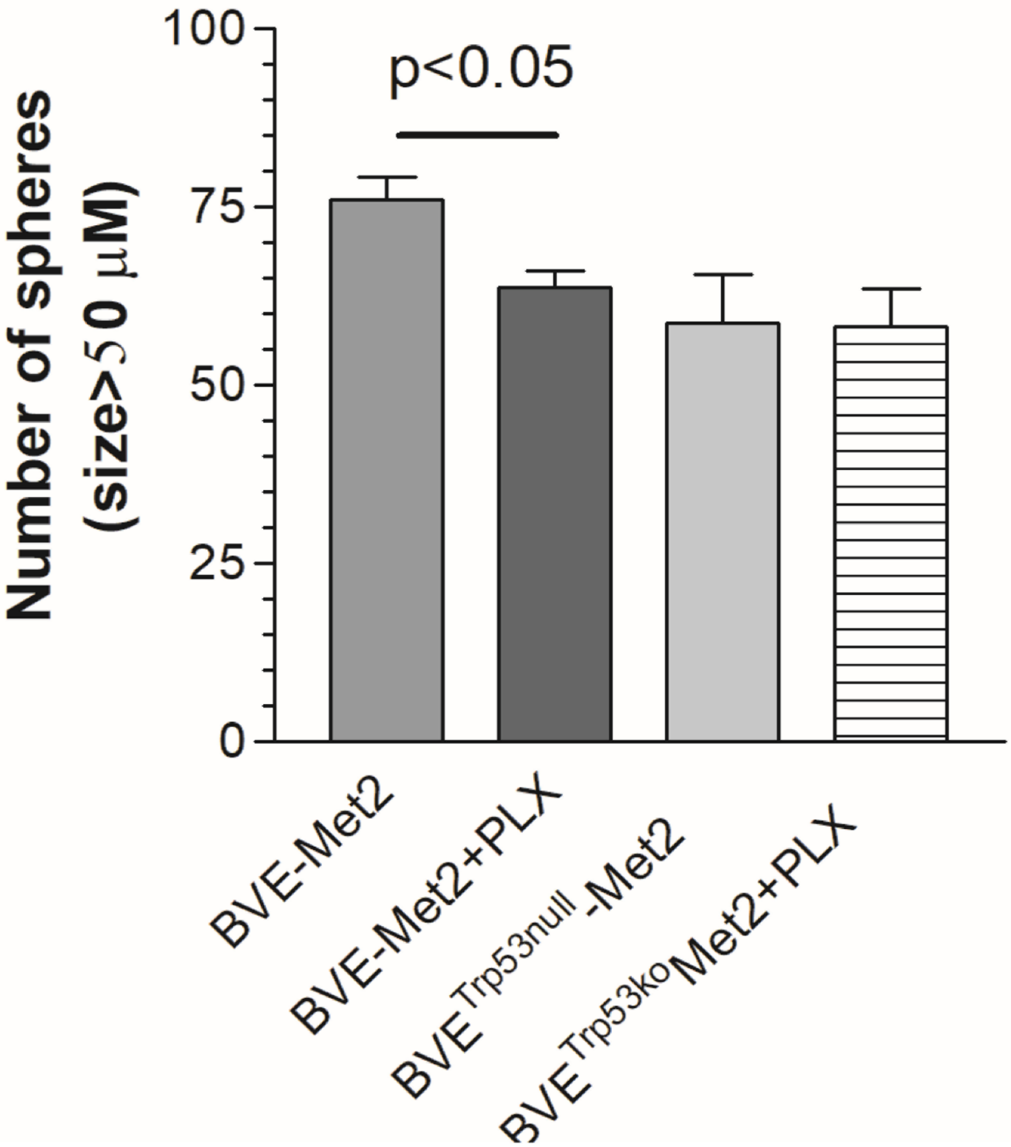

Supplementary Figure 4. Effect of BRAF^{V600E} inhibitor PLX4720 on tumorsphere formation. Reduction (10%) of tumorsphere formation was observed in BVE-Met2 cells after 2μM PLX4720 incubation. No significant inhibition was found in BVE^{Trp53null}-Met2 cells treated with 2μM PLX4720.

Supplementary Table 1. 110 DEGs present in all Met2 cell lines

Gene ID	Gene Symbol	KGD-Met2	KGD^{CDKN2A_null}-Met2	BVE-Met2	BVE^{Trp53_null}-Met2	BVE^{Trp53_null}-Met2
11421	Ace	4.445818619	6.163230349	2.321928095	7.592457037	9.269126679
13733	Adgre1	3.584962501	8.224001674	11.02097994	10.02928723	7.22881869
107747	Aldh1l1	8.596189756	3.680857928	3.378511623	7.294620749	4.584962501
11690	Alox5ap	5.285402219	4.834576391	5.994579724	12.09242682	9.016808288
105827	Amigo2	3.906890596	5.321928095	8.044394119	4.087462841	8.108524457
77531	Anks1b	2.475733431	5.523561956	3.357552005	3.672425342	3.087462841
75761	Apol7a	3.421463768	2.5360529	4.754887502	3.321928095	2.925999419
11846	Arg1	5.392317423	11.55650605	11.2179577	15.65079933	11.85018684
216445	Arhgap9	5.209453366	6.64385619	6.725001003	8.056637715	5.022367813
219144	Arl11	2.30580843	3.902512155	8.670656249	5.317593505	2.526068812
74008	Arsg	2.099535674	5.209453366	4.280107919	5.345774837	3.584962501
12259	C1qa	4.64385619	9.231221181	11.57695666	12.49660415	10.49884921
12351	Car4	4.321928095	10.6926155	10.52552081	8.511752654	7.303780748
108723	Card11	2.584962501	4.459431619	7.312882955	5.491853096	3.906890596
12424	Cck	5.977279923	8.21916852	5.7642862	12.64069684	10.33650656
20302	Ccl3	5.196397213	10.43879185	13.10901442	12.06676193	9.682994584
20305	Ccl6	4.66106548	12.2822197	14.45545566	11.40118722	7.84862294
60533	Cd274	2.622437206	4.392317423	5.080373416	6.453956489	4.087462841
23833	Cd52	5.700439718	7.714245518	12.10394343	12.76155123	9.350939182
17064	Cd93	5.087462841	2	4.353636955	5.260235772	2.294834434
56620	Clec4n	3.070389328	11.68606275	13.83249448	13.46454575	10.59058705
170571	Cntnap4	5.087462841	4.247927513	4.584962501	8.523561956	8.326429487
12835	Col6a3	9.285402219	3.499146534	11.37123213	6.95419631	7.451211112
12978	Csf1r	4.087462841	2.350497247	8.543805176	6.261423356	3.727364824
12983	Csf2rb	2.263034406	4.341219433	11.56938096	10.68270204	8.080373416
13040	Ctss	2.142957954	10.90162116	12.90388185	12.87670878	9.082149041
13058	Cybb	4.700439718	7.21916852	10.07146236	10.36632221	6.523561956
227929	Cytip	4.311586151	5.357552005	6.392317423	7.276124405	6.554588852
13586	Ear1	5.491853096	10.07146236	12.15323534	11.7232343	8.599912842
503845	Ear12	6.942514505	10.72109919	12.60894781	12.27699674	9.337621902
13587	Ear2	5.672425342	10.2467406	11.62067804	10.30947635	7.826548487
66892	Eif4e3	3.44625623	2.20469042	5.247927513	3.235216462	2.142957954
14127	Fcer1g	7.90000421	5.064718273	9.735146536	13.74336181	11.21067134
246256	Fcgr4	4.523561956	5.562242424	9.968666793	9.977279923	5.807354922
14182	Fgfr1	3.72377697	2.20650938	4.237669608	2.573841461	3.395694503
14652	Glp1r	7.857980995	5.709658248	5.523561956	8.409390936	10.59339112
217122	Gm11545	4.247927513	5.087462841	4.539158811	7.62935662	4.64385619
93695	Gpnmb	4.523561956	12.12734954	13.42232762	10.70090649	8.292498618
106512	Gpsm3	2.791099031	2.797484678	6.627821838	4.821880254	2.600904045
68713	Ifitm1	6.794415866	3.855556767	5.682278733	9.923327485	6

16012	Igfbp6	7.111464155	6.212574646	7.982845454	3.242428034	3.916706849
16170	Il16	3.169925001	5.357552005	9.95419631	6.554588852	3.169925001
16181	Il1rn	5.129283017	3	4.320223117	5.689868588	2.68182404
16186	Il2rg	7.95419631	3.215874587	10.22400167	5.828990836	2.700439718
16331	Inpp5d	4.087462841	3.491853096	3.275634443	9.328674927	6.977279923
15900	Irf8	4.481557281	2.396475048	4.240746339	9.418455019	9.071462363
16414	Itgb2	2.102361718	6.034423831	5.863118194	5.551758848	2.688710426
223272	Itgbl1	5.106915204	4.156504486	8.871135184	9.194756854	6.857980995
16792	Laptm5	8.900866808	11.28915435	11.55090708	13.23187117	10.36996079
77889	Lbh	2.144920995	2.385493224	3.066790087	3.327173397	2.882481103
18826	Lcp1	4.95419631	8.199672345	9.262094845	12.87152026	10.15608308
14728	Lilrb4a	6.426264755	4.487265827	4.84434913	6.355859296	2.612443237
107321	Lpxn	3.879145605	6.807354922	4.745954377	6.925124944	4.490164746
74511	Lrrc17	5.491853096	6.820178962	4.807354922	3.584962501	4.906890596
17105	Lyz2	3.144771524	15.92942024	12.28517207	18.38606035	14.72818626
74490	Mamstr	5.781359714	4.283383051	3.938599455	4.857980995	5.169925001
17167	Marco	4.169925001	11.91251514	9.991135289	4.599912842	4.209453366
68279	Mcoln2	2.05626822	3.700439718	5.614709844	5.832890014	4.247927513
17289	Mertk	4.372554168	3.584962501	4.285402219	2.159198595	2.736965594
17381	Mmp12	4.68182404	4.851749041	9.917869303	13.25841881	10.53138146
68774	Ms4a6d	4.459431619	7.971543554	10.74399286	10.98726401	8.055282436
17916	Myo1f	2.807354922	7.906890596	10.19967234	10.49785184	8.383704292
17969	Ncf1	2.807354922	7.554588852	10.07012094	9.063395081	6.132713922
105855	Nckap1	5.426264755	4.437405312	9.638435914	10.3858624	7.876516947
407790	Ndufa4l2	3.911357147	5.882643049	5.614709844	10.6183855	10.72280753
216799	Nlrp3	4.392317423	5.058893689	9.027905997	8.918863237	6.50779464
107607	Nod1	5.604862058	2.512166675	7.22881869	4.541373232	2.192645078
224109	Nrros	3	7.599912842	8.087462841	5.765126687	3.81526012
14726	Pdpn	2.187255581	7	5.882319139	11.94727165	8.515699838
218194	Phactr1	2.95419631	3.027480736	2.807354922	4.357552005	4.044394119
104759	Pld4	4.307428525	2.839959587	11.3858624	9.205060111	5.87282876
235527	Plscr4	3.150941898	2.538419915	6.539158811	4.64385619	4.459431619
72324	Plxdc1	7.357552005	4.619608644	6.548436625	4.14974712	3.285402219
19222	Ptgir	3.459431619	3.155278225	5.683696454	7.044394119	4.459431619
19731	Rgl1	2.318698007	2.667855509	4.061296179	5.800703842	4.949251941
380713	Scarf1	3.169925001	5.375039431	7	5.392317423	2.938599455
58234	Shank3	2.271596419	2.717856771	6.06608919	4.022367813	5.184875343
20612	Siglec1	2.807354922	7.118941073	10.2632692	8.965784285	5.523561956
620235	Siglec15	2.38466385	3.654864514	4.56193706	2.339850003	4.430452552
65221	Slc15a3	4.058893689	2.450056088	11.85018684	11.71467483	10.04302728
13602	Sparcl1	4.431845787	4.392317423	10.41785251	5.614709844	6.845490051
21391	Tbxas1	5.357552005	7.894817763	9.224001674	10.32755264	7.303780748
23965	Tenm3	7.163901214	3	5.807354922	5.569855608	4.922832139
23966	Tenm4	3.459431619	5	2.807354922	3.218423519	3.302375297

21926	Tnf	4.169925001	3.574908836	8.977279923	3.972692654	2.510961919
22041	Trf	5.906890596	5.071587082	11.58824615	13.98708633	9.259743264
22376	Was	2.632268215	3.886132035	9.177419538	6.037089319	3.039528364
100034251	Wfdc17	8.164906927	8.583082768	14.81217731	11.87651695	8.596189756
74328	1700047E10Rik	-3.10433666	-2.511819645	-2.203807686	-2.838381818	-4.361943774
71874	2310007B03Rik or Mab21L4	-4.350497247	-8.768184325	-10.72536626	-12.34346334	-11.34346334
57278	Bcam	-2.295092622	-4.565257091	-7.58954712	-6.451623252	-6.882780416
67445	C1qtnf4	-5.426264755	-6.456149035	-6.129283017	-5.028408415	-3.843983844
66371	Chmp4c	-2.561878888	-5.459431619	-5.390407833	-5.501439145	-10.82336724
12804	Cntfr	-5.584962501	-6.108524457	-5.934673752	-3.361456459	-4.94641896
12829	Col4a4	-2.550197083	-3.700439718	-5.033423002	-2.892570924	-6.325530332
13106	Cyp2e1	-3.96212482	-5.842350343	-7.328674927	-2.469726414	-2.043681924
14073	Faah	-3.584962501	-4.459431619	-6.894817763	-5.205548911	-6.527477006
64339	Fndc4	-4.340368678	-6.222112368	-2.782233809	-2.496874538	-2.244790432
319167	Hist1h2ag	-8.654636029	-4.312239804	-2.342035668	-7.434628228	2.255369744
17388	Mmp15	-5.285402219	-6.209453366	-5.194756854	-7.436711542	-6.851749041
50873	Park2	-11.77941158	-3.169003667	-4.921570491	-8.161111227	-4.207678046
18510	Pax8	-3.321928095	-4.321928095	-7.577428828	-4.024946357	-6.451211112
19208	Ptcra	-4.182203331	-2.574470127	-4.135159583	-2.7589919	-4.343954401
19268	Ptprf	-2.775473414	-3.147643554	-4.840761007	-5.06463135	-4.557671361
72433	Rab38	-5.459431619	-2.347923303	-7.082149041	-4.380821784	-7.965784285
234214	Sorbs2	-5.101538026	-5.129283017	-4.584962501	-8.586214297	-8.586214297
20666	Sox11	-6.189824559	-3.029747343	-4.584962501	-7.108524457	-7.108524457
66260	Tmem54	-4.614709844	-4.26497221	-11.00457162	-12.31061278	-9.14068778
21912	Tspan7	-8.988684687	-9.299208018	-2.523754946	-5.849162407	-5.824500353
665976	Vmn2r-ps129	-3.641344971	-2.007494537	-2.648288436	-3.469114172	-3.317111079

Supplementary Table 2. Hub genes in thyroid cancer metastasis

	Gene symbol (degree of node)	Description	FDA approved drug targets	Biological role in cancer	Reference
1	Tnf (47)	Tumor Necrosis Factor	Etanercept, Infliximab, Certolizumab, Adalimumab	A multifunctional proinflammatory cytokine. Involved in the regulation of a wide spectrum of biological processes including cell proliferation, differentiation, apoptosis, lipid metabolism, and coagulation. High TNF levels impede tumor growth and low levels would promote cancer development and progression	(1)
2	Fgfr1 (46)	Fibroblast Growth Factor Receptor 1	Lenvatinib (multikinase inhibitor, inhibiting FGFR1-4, VEGFR1-3, RET, KIT and PDGFR-β)	A member of the fibroblast growth factor receptor family of receptor tyrosine kinases, and functions mainly via PI3K and MAPK pathways. It promotes cell proliferation, epithelial–mesenchymal transition, and metastasis via FGFR1-ERK1/2-SOX2 axis.	(2)
3	Was (30)	WASP Actin Nucleation Promoting Factor	NA	Wiskott-Aldrich Syndrome protein (WASp) is an actin nucleation-promoting factor and is a key regulator of actin polymerization involved in cancer metastasis.	(3,4)
4	Itgb2 (27)	Integrin Subunit Beta 2	NA	heterodimeric leukocyte adhesion molecule essential for adhesion, trafficking and T cell effector function. Expression in cancer cells promotes invasion and metastasis in a manner mimicking leukocytes via YAP1, a downstream nuclear effector of the Hippo signaling pathway and PRDM4	(5)
5	Ncf1 (25)	Neutrophil Cytosolic Factor 1	NA	a cytosolic component of the NADPH oxidase 2 (NOX2) complex required for the	(6)

				production of reactive oxygen species (ROS), which promotes metastatic colonization	
6	Fcer1g (20)	Fc Epsilon Receptor Ig	NA	a key molecule involved in allergic inflammatory reactions. Up-regulated in most tumour and closely related to tumour microenvironment and tumour immunity. High expression of FCER1G in ccRCC is closely related to TAMs infiltration and suppression of T cell activation and proliferation. FCER1G-associated partners were enriched in pathways associated with neutrophils activation	(7)
7	C1qa (19)	Complement C1q A Chain	NA	C1q is the activator of the classical complement pathway. C1q acts in the tumour microenvironment as a cancer-promoting factor unrelated to complement activation	(8,9)
8	Inpp5d (19)	Inositol Polyphosphate-5-Phosphatase D or<br><br>Src homology 2 domain containing inositol polyphosphate 5-phosphatase 1 (SHIP1)	NA	Promote cancer cell survival, metastasis and growth by enhancing AKT activation through production of PI(3,4)P2	(10)
9	Il2rg (18)	Interleukin 2 Receptor Subunit Gamma	NA	an important cytokine receptor sub-unit that is common to at least six different interleukin receptors: IL-2, IL-4, IL-7, IL-9, IL-15 and interleukin-21 receptor. Its overexpression mediates cancer cell growth through the JAK/Stat pathway	(11)

10	Shank3 (15)	SH3 And Multiple Ankyrin Repeat Domains 3	NA	A synaptic scaffolding protein and a regulator of integrin activity. Act as integrin activation inhibitors by sequestering active Rap1. SHANK3 silencing triggers increased plasma membrane Rap1 activity, cell spreading, migration and invasion.<br><br>Shank3 protein mediates sustained Erk–MAPK and PI3K signaling in epithelial cells<br><br>A novel tumor suppressor that binds to overactive mutant KRAS to limit oncogenic KRAS signaling and maintain RAS activity at an optimal level. SHANK3 directly interacts with KRAS and competes with RAF for KRAS binding to sustain oncogenic RAS-MAPK/ERK signalling at an optimal level (i.e. below toxic oncogenic signalling) in KRAS-mutant cancers	(12,13)<br><br>https://www.biorxiv.org/content/10.1101/2022.09.21.508660v1.full.pdf
11	Nckap1<br><br>(13)	NCK Associated Protein 1	NA	Part of WASF regulatory complex including CYFIP1, NCKAP1, ABI1 and BRK1. Promote metastasis by regulating EMT	(14,15)
12	Nod1 (13)	Nucleotide Binding Oligomerization Domain Containing 1	NA	A cytoplasmic pattern recognition receptor. Its activation augments cancer cell adhesion, migration and metastasis via p38 mitogen activated protein kinase (MAPK) pathway. Nod1 and Nod2 activation initiates a pro-inflammatory<br><br>response downstream of the nuclear factor kB (NF-kB) and<br><br>mitogen-activated protein kinase (MAPK) pathways	(16,17)

13	Card11 (12)	Caspase Recruitment Domain Family Member 11	NA	A scaffold protein specifically interacting with BCL10 to activate NF-κB. Aberrant NF-κB activation leads to lymphoma and skin cancer. It also activates the mTOR pathway in lung and renal cell carcinoma, resulting in suppressed autophagy.	(18,19)
14	Csf2rb (12)	Colony Stimulating Factor 2 Receptor Subunit Beta	NA	CSF2RB; CD131) is the common subunit of the type I cytokine receptors for granulocyte-macrophage colony-stimulating factor (GM-CSF), interleukin (IL)-3 and IL-5. FOXP3+ regulatory T cells (Tregs) highly overexpress CSF2RB. Involved in pro-inflammatory TAM activation in brain metastasis.	(20)
15	Cybb (11)	Cytochrome B-245 Beta Chain	NA	A primary component of the microbicidal oxidase system of phagocytes that generates superoxide or reactive oxygen species (ROS). Genetic depletion of any of the NOX2 subunits Cyba, Cybb, Ncf1, Ncf2 and Ncf4 reduced the formation of lung metastases	(21)
16	Fcgr4 (11)	Fc receptor, IgG, low affinity IV that is closely related to human FCGR3A (Fc gamma RIIIA , 60% amino acid identity)	NA	Enables IgE receptor activity and IgG receptor activity. Involved in neutrophil activation and tumor immunity. High expression of FCGR3A was associated with poor prognosis for patients with prostate cancer	(22,23)
17	Nlrp3 (11)	NLR Family Pyrin Domain Containing 3	NA	The NLRP3 inflammasome is a multimeric cytosolic protein complex and functions as an upstream activator of NF-kappaB signaling. It plays a role in the regulation of inflammation, immune response, and apoptosis. It is	(24)

				involved in the activation of caspase-1, leading to secretion of inflammatory cytokines IL1B and IL18 and inflammatory cell death, pyroptosis.	
18	Csf1r (10)	Colony Stimulating Factor 1 Receptor	Pexidartinib (PLX3397)	Intratumor CSF-1/CSF-1R signaling is known to be overexpressed in many tumor types and associated with poor prognosis. Its activation causes recruitment of TAMs and development of pro-tumor inflammatory environment, leading to tumor growth and metastasis	(25,26)
19	Irf8 (272)	Interferon regulatory factor 8	NA	a transcription factor of the interferon regulatory factor family and functions as tumor suppressor	(27)

Supplementary References

1. Montfort A, Colacios C, Levade T, Andrieu-Abadie N, Meyer N, Ségui B. The TNF Paradox in Cancer Progression and Immunotherapy. *Frontiers in immunology*. 2019;10:1818.
2. Wang K, Ji W, Yu Y, Li Z, Niu X, Xia W, Lu S. FGFR1-ERK1/2-SOX2 axis promotes cell proliferation, epithelial–mesenchymal transition, and metastasis in FGFR1-amplified lung cancer. *Oncogene*. 2018;37(39):5340-5354.
3. Lane J, Martin T, Weeks HP, Jiang WG. Structure and role of WASP and WAVE in Rho GTPase signalling in cancer. *Cancer genomics & proteomics*. 2014;11(3):155-165.
4. Biber G, Ben-Shmuel A, Noy E, Joseph N, Puthenveetil A, Reiss N, Levy O, Lazar I, Feiglin A, Ofran Y, Kedmi M, Avigdor A, Fried S, Barda-Saad M. Targeting the actin nucleation promoting factor WASp provides a therapeutic approach for hematopoietic malignancies. *Nature communications*. 2021;12(1):5581.
5. Liu H, Dai X, Cao X, Yan H, Ji X, Zhang H, Shen S, Si Y, Zhang H, Chen J, Li L, Zhao JC, Yu J, Feng XH, Zhao B. PRDM4 mediates YAP-induced cell invasion by activating leukocyte-specific integrin β2 expression. *EMBO reports*. 2018;19(6).
6. Zhong J, Li Q, Luo H, Holmdahl R. Neutrophil-derived reactive oxygen species promote tumor colonization. *Communications biology*. 2021;4(1):865.
7. Yang R, Chen Z, Liang L, Ao S, Zhang J, Chang Z, Wang Z, Zhou Y, Duan X, Deng T. Fc Fragment of IgE Receptor Ig (FCER1G) acts as a key gene involved in cancer immune infiltration and tumour microenvironment. *Immunology*. 2023;168(2):302-319.
8. Bulla R, Tripodo C, Rami D, Ling GS, Agostinis C, Guarnotta C, Zorzet S, Durigutto P, Botto M, Tedesco F. C1q acts in the tumour microenvironment as a cancer-promoting factor independently of complement activation. *Nature communications*. 2016;7:10346.
9. Roumenina LT, Daugan MV, Noé R, Petitprez F, Vano YA, Sanchez-Salas R, Becht E, Meilleroux J, Clec'h BL, Giraldo NA, Merle NS, Sun CM, Verkarre V, Validire P, Selves J, Lacroix L, Delfour O, Vandenberghe I, Thuilliez C, Keddani S, Sakhi IB, Barret E, Ferré P, Corvaïa N, Passioukov A, Chetaille E, Botto M, de Reynies A, Oudard SM, Mejean A, Cathelineau X, Sautès-Fridman C, Fridman WH. Tumor Cells Hijack Macrophage-

Produced Complement C1q to Promote Tumor Growth. *Cancer immunology research*. 2019;7(7):1091-1105.
10. Pedicone C, Meyer ST, Chisholm JD, Kerr WG. Targeting SHIP1 and SHIP2 in Cancer. *Cancers*. 2021;13(4).
11. Ayars M, O'Sullivan E, Macgregor-Das A, Shindo K, Kim H, Borges M, Yu J, Hruban RH, Goggins M. IL2RG, identified as overexpressed by RNA-seq profiling of pancreatic intraepithelial neoplasia, mediates pancreatic cancer growth. *Oncotarget*. 2017;8(48):83370-83383.
12. Lilja J, Zacharchenko T, Georgiadou M, Jacquemet G, De Franceschi N, Peuhu E, Hamidi H, Pouwels J, Martens V, Nia FH, Beifuss M, Boeckers T, Kreienkamp HJ, Barsukov IL, Ivaska J. SHANK proteins limit integrin activation by directly interacting with Rap1 and R-Ras. *Nature cell biology*. 2017;19(4):292-305.
13. Schuetz G, Rosário M, Grimm J, Boeckers TM, Gundelfinger ED, Birchmeier W. The neuronal scaffold protein Shank3 mediates signaling and biological function of the receptor tyrosine kinase Ret in epithelial cells. *The Journal of cell biology*. 2004;167(5):945-952.
14. Kwon MR, Lee JH, Park J, Park SS, Ju EJ, Ko EJ, Shin SH, Son GW, Lee HW, Kim YJ, Song SY, Jeong S-Y, Choi EK. NCK-associated protein 1 regulates metastasis and is a novel prognostic marker for colorectal cancer. *Cell Death Discovery*. 2023;9(1):7.
15. Teng Y, Qin H, Bahassan A, Bendzunas NG, Kennedy EJ, Cowell JK. The WASF3-NCKAP1-CYFIP1 Complex Is Essential for Breast Cancer Metastasis. *Cancer research*. 2016;76(17):5133-5142.
16. Jiang HY, Najmeh S, Martel G, MacFadden-Murphy E, Farias R, Savage P, Leone A, Roussel L, Cools-Lartigue J, Gowing S, Berube J, Giannias B, Bourdeau F, Chan CHF, Spicer JD, McClure R, Park M, Rousseau S, Ferri LE. Activation of the pattern recognition receptor NOD1 augments colon cancer metastasis. *Protein & cell*. 2020;11(3):187-201.
17. Maisonneuve C, Tsang DKL, Foerster EG, Robert LM, Mukherjee T, Prescott D, Tattoli I, Lemire P, Winer DA, Winer S, Streutker CJ, Geddes K, Cadwell K, Ferrero RL, Martin A, Girardin SE, Philpott DJ. Nod1 promotes colorectal carcinogenesis by regulating the immunosuppressive functions of tumor-infiltrating myeloid cells. *Cell reports*. 2021;34(4):108677.
18. McGuire MH, Dasari SK, Yao H, Wen Y, Mangala LS, Bayraktar E, Ma W, Ivan C, Shoshan E, Wu SY, Jonasch E, Bar-Eli M, Wang J, Baggerly KA, Sood AK. Gene Body Methylation of the Lymphocyte-Specific Gene CARD11 Results in Its Overexpression and Regulates Cancer mTOR Signaling. *Molecular cancer research : MCR*. 2021;19(11):1917-1928.
19. Watt SA, Purdie KJ, den Breems NY, Dimon M, Arron ST, McHugh AT, Xue DJ, Dayal JH, Proby CM, Harwood CA, Leigh IM, South AP. Novel CARD11 Mutations in Human Cutaneous Squamous Cell Carcinoma Lead to Aberrant NF-κB Regulation. *The American journal of pathology*. 2015;185(9):2354-2363.
20. Klemm F, Möckl A, Salamero-Boix A, Alekseeva T, Schäffer A, Schulz M, Niesel K, Maas RR, Groth M, Elie BT, Bowman RL, Hegi ME, Daniel RT, Zeiner PS, Zinke J, Harter PN, Plate KH, Joyce JA, Sevenich L. Compensatory CSF2-driven macrophage activation promotes adaptive resistance to CSF1R inhibition in breast-to-brain metastasis. *Nature cancer*. 2021;2(10):1086-1101.
21. Martner A, Aydin E, Hellstrand K. NOX2 in autoimmunity, tumor growth and metastasis. *The Journal of pathology*. 2019;247(2):151-154.
22. Zha Z, Hong Y, Tang Z, Du Q, Wang Y, Yang S, Wu Y, Tan H, Jiang F, Zhong W. FCGR3A: A new biomarker with potential prognostic value for prostate cancer. *Frontiers in oncology*. 2022;12:1014888.
23. Li L, Huang Z, Du K, Liu X, Li C, Wang D, Zhang Y, Wang C, Li J. Integrative Pan-Cancer Analysis Confirmed that FCGR3A is a Candidate Biomarker Associated With Tumor Immunity. *Frontiers in pharmacology*. 2022;13:900699.
24. Sharma BR, Kanneganti TD. NLRP3 inflammasome in cancer and metabolic diseases. *Nature immunology*. 2021;22(5):550-559.
25. Patsialou A, Wyckoff J, Wang Y, Goswami S, Stanley ER, Condeelis JS. Invasion of human breast cancer cells in vivo requires both paracrine and autocrine loops involving the colony-stimulating factor-1 receptor. *Cancer research*. 2009;69(24):9498-9506.
26. Ryder M, Gild M, Hohl TM, Pamer E, Knauf J, Ghossein R, Joyce JA, Fagin JA. Genetic and pharmacological targeting of CSF-1/CSF-1R inhibits tumor-associated macrophages and impairs BRAF-induced thyroid cancer progression. *PloS one*. 2013;8(1):e54302.

27. Yang D, Thangaraju M, Greeneltch K, Browning DD, Schoenlein PV, Tamura T, Ozato K, Ganapathy V, Abrams SI, Liu K. Repression of IFN regulatory factor 8 by DNA methylation is a molecular determinant of apoptotic resistance and metastatic phenotype in metastatic tumor cells. *Cancer research*. 2007;67(7):3301-3309.
